# Exosome-transported circ_0061407 and circ_0008103 play a tumour-repressive role and show diagnostic value in non-small-cell lung cancer

**DOI:** 10.1186/s12967-024-05215-6

**Published:** 2024-05-06

**Authors:** Zhenhua Chen, Xinyi Ma, Ziyuan Chen, Wei Chen, Leyi Li, Yichen Lin, Yulin Hu, Yue Shang, Yikai Zhao, Jinxian He, Chengwei Zhou, Xiaodan Meng

**Affiliations:** 1 Department of Biochemistry and Molecular Biology, School of Basic Medical Sciences, Health Science Center, Ningbo University, 818 Fenghua Road, Ningbo, 315211 Zhejiang China; 2 Zhejiang Provincial Key Laboratory of Pathophysiology, Health Science Center, Ningbo University, Ningbo, 315211 Zhejiang China; 3 Department of Thoracic Surgery, The First Affiliated Hospital of Ningbo University, Ningbo, 315020 Zhejiang China; 4 Department of Thoracic Surgery, The Ningbo Medical Center Lihuili Hospital, Ningbo University, Ningbo, 315048 China

**Keywords:** Exosome, Circular RNA, Non-small-cell lung cancer, Tumour marker

## Abstract

**Background:**

Circular RNAs (circRNAs), one of the major contents of exosomes, have been shown to participate in the occurrence and progression of cancers. The role and the diagnostic potential of exosome-transported circRNAs in non-small-cell lung cancer (NSCLC) remain largely unknown.

**Methods:**

The NSCLC-associated exosomal circ_0061407 and circ_0008103 were screened by circRNA microarray. The role of circ_0061407 and circ_0008103 in NSCLC was examined in vitro and in vivo. The encapsulation of the two circRNAs into exosomes and the transport to recipient cells were observed by confocal microscopy. The effects of exosome-transported circ_0061407 and circ_0008103 on recipient cells were investigated using a co-culture device. Bioinformatics analyses were performed to predict the mechanisms by which circ_0061407 and circ_0008103 affected NSCLC. The quantitative polymerase chain reaction was used to quantify the exosome-containing circ_0061407 and circ_0008103 in the serum samples of healthy, pneumonia, benign lung tumours, and NSCLC. The diagnostic efficacy was evaluated using receiver operating characteristic curves.

**Results:**

The levels of circ_0061407 and circ_0008103 within exosomes were down-regulated in the serum of patients with NSCLC. The up-regulation of circ_0061407 and circ_0008103 inhibited the proliferation, migration/invasion, cloning formation of NSCLC cells in vitro and inhibited lung tumour growth in vivo. Circ_0061407 and circ_0008103 were observed to be packaged in exosomes and transported to recipient cells, where they inhibited the proliferation, migration/invasion, and cloning formation abilities of the recipient cells. Moreover, circ_0061407 and circ_0008103 might be involved in the progression of NSCLC by interacting with microRNAs and proteins. Additionally, lower serum exosomal circ_0061407 and circ_0008103 levels were associated with advanced pathological staging and distant metastasis.

**Conclusions:**

This study identified two novel exosome-transported circRNAs (circ_0061407 and circ_0008103) associated with NSCLC. These findings may provide additional insights into the development of NSCLC and potential diagnostic biomarkers for NSCLC.

**Supplementary Information:**

The online version contains supplementary material available at 10.1186/s12967-024-05215-6.

## Background

Lung cancer is the most prevalent malignant tumour with the highest morbidity and mortality worldwide [1]. Non-small-cell lung cancer (NSCLC) accounts for 85% of all lung cancer cases [2]. Lung cancer does not exhibit clear symptoms at early stages and patients are usually diagnosed at later stages, losing the opportunity to accept operation. Many treatments, including chemotherapy, radiotherapy, immunotherapy, molecular-targeted therapy, and so on, are available for patients. The five-year survival rate of lung cancer is less than 20% [3]. Therefore, exploring the mechanisms of lung cancer-related molecules involved in the occurrence and progression of lung cancer, and discovering novel diagnostic markers for lung cancer are of great significance to improve patient survival [4].

Exosomes are small extracellular vesicles that range in diameter from 30 to 200 nm. They have a lipid bilayer structure which protects the contents from the digestion of nucleases and proteases. Exosomes contain various biologically active substances, including circular RNA (circRNA), microRNA (miRNA), long non-coding RNA (lncRNA), messenger RNA (mRNA), DNA, and proteins [5–7]. These bioactive substances are carried by exosomes and released into the extracellular environment. Recipient cells recognize and take up these substances, which then affect the physiological and pathological functions of recipient cells [8]. In this way, exosomes are extensively involved in intercellular communication and play a crucial role in diseases, in particular in cancers [9]. Studies have shown that exosomes are participated in tumour growth, apoptosis, angiogenesis, epithelial-mesenchymal transition (EMT), and metastasis [10, 11]. Exosomes are also involved in immune evasion and chemotherapeutic drug resistance in tumours [12–14].

CircRNAs are the most abundant non-coding RNAs (ncRNAs) in exosomes. CircRNAs are closed-loop RNA transcripts formed by a special splicing manner termed back-splicing. Briefly, circRNAs covalently link the 3’-end of an exon to the 5’-end of its own or an upstream exon to form a closed structure with a back-splicing junction site [15, 16]. CircRNAs have diverse functions through a variety of mechanisms. They act as miRNA sponges by competitively binding to miRNAs, leading to the derepression of miRNAs on the target mRNAs [17]. Additionally, they function as transcriptional and splicing regulators through binding to RNA-binding proteins [18]. Intriguingly, some circRNAs can serve as templates for polypeptide synthesis [19, 20]. Based on their regulatory functions, circRNAs have been reported to play an essential role in diseases, especially in cancers. For example, circRNA BIRC6 promotes NSCLC progression by sponging miR-145 [21]. CircRNA VMP1 induced cisplatin resistance in NSCLC by targeting the miR-524-5p/METTL3/SOX2 axis [22]. Additionally, circRNA SCAP interacts with splicing factor 3a subunit 3 and inhibits NSCLC progression by activating p53 signaling transduction [23].

Considering the important role of exosomes and circRNAs in NSCLC, we designed the present study to uncover novel NSCLC-related circRNAs incorporated in exosomes. Two down-regulated circRNAs, circ_0008103 and circ_0061407 were identified using circRNA array and quantitative polymerase chain reaction (qPCR). We observed the encapsulation of circ_0008103 and circ_0061407 into exosomes and their release to the cytoplasm of recipient cells. Also, we found that circ_0008103 and circ_0061407 could be transported by exosomes to suppress the proliferation, migration, and invasive abilities of recipient cells. We further measured the levels of exosomal circ_0008103 and circ_0061407 in the serum samples from healthy individuals, patients with pneumonia, patients with benign lung tumours, and patients with NSCLC. We found that the levels of serum exosomal circ_0008103 and circ_0061407 were significantly decreased in NSCLC cohorts compared to the healthy, pneumonia, and benign lung tumour cohorts. And low levels of serum exosomal circ_0061407 and circ_0008103 were associated with advanced tumour stages and distant metastasis in NSCLC. The bioinformatic analyses revealed the potential mechanisms by which circ_0008103 and circ_0061407 might be involved in the development of lung cancer. Collectively, we identified two tumour-suppressive circRNAs in exosomes, which might serve as potential diagnostic and therapeutic targets for NSCLC.

## Methods

### Clinical samples

Serum samples were randomly selected from patients with NSCLC (n = 122), benign lung tumours (n = 20), and pneumonia (n = 40) at the first diagnosis and before surgery or treatment in the First Affiliated Hospital of Ningbo University (Ningbo, China) and Li Huili Hospital Affiliated to Ningbo University (Ningbo, China) from 2020 to 2022. Forty-six serum samples from healthy individuals with no history of tumours and other diseases were taken from Ningbo Kangning Hospital (Ningbo, China). In this study, each subject filled in an informed consent form about the collection of his/her blood for research. The blood collection was approved by the Clinical Research Ethics Committee of the Health Science Center of Ningbo University, and in accordance with the principles of the Declaration of Helsinki. The clinical characteristics (age, gender, smoking history, histological subtype, TNM stage, lymph node metastasis, distant metastasis, tumour size, etc.) of all the patients and the clinical data about healthy volunteers involved in the study are shown in Table 1.

**Table 1:** The serum samples and the corresponding clinical parameters in the present study

Sample types and clinical data	Numbers n(%)
Non-small-Cell Lung Cancer	122 (100)
Age(year)	
< 60	107 (87.7)
≥ 60	15 (12.3)
Gender	
Male	37 (30.3)
Female	85 (69.7)
Smoking	
Yes	51 (41.8)
No	71 (58.2)
Tumor size(cm)	
≤ 5	75 (61.5)
> 5	39 (32.0)
Unknown	8 (6.5)
Histologic subtypes	
Squamous cell carcinoma	66 (54.1)
Adenocarcinoma	56 (45.9)
TNM stage	
I	12 (9.8)
II	35 (28.7)
III	44 (36.1)
IV	31 (25.4)
Distant metastasis	
M0	91 (74.6)
M1	31 (25.4)
Lymph node metastasis	
N0	23 (18.9)
N1-3	97 (79.5)
Unknown	2 (1.6)
Pneumonia	40 (100)
Age(year)	
< 60	37 (92.5)
≥ 60	3 (7.5)
Gender	
Male	10 (25.0)
Female	30 (75.0)
Benign lung tumor	20 (100)
Age(year)	
< 60	16 (80.0)
≥ 60	4 (20.0)
Gender	
Male	10 (50.0)
Female	10 (50.0)
Healthy	46 (100)
Age(year)	
< 60	35 (76.1)
≥ 60	11 (23.9)
Gender	
Male	8 (17.4)
Female	38 (82.6)

### Cell culture and cell transfection

All cell lines, including NCI-H1299, A549, SPC-A-1, LTEP-A2, and Beas-2B were purchased from Cell Bank/Stem Cell Bank (Shanghai, China). The human normal lung epithelial cell line Beas-2B was cultured in DMEM basal medium (Corning, USA). The human NSCLC cell lines NCI-H1299, A549, SPC-A-1, and LTEP-A2 were cultured using RPMI-1640 basal medium (Corning, USA). The above basal culture medium was supplemented with 10% fetal bovine serum (FBS; PAN, Germany), 100-μg/mL penicillin, and 100-U/mL streptomycin (New Cell & Molecular Biotech, USA). Cells were cultured in a humidified incubator at 37 °C with 5% CO_2_. Exosomes were removed from FBS at 110,000 × g, 4 °C, for 8 h by an ultracentrifuge device (BECKMAN COULTER, USA) as previously described. The Lipofectamine 2000 reagent (Invitrogen, USA) was used to deliver the recombinant plasmids into cells as previously reported [2].

### Vector construction

To investigate the role of circRNAs (circ_0061407 and circ_0008103) in NSCLC, we constructed overexpression plasmids of circ_0061407 and circ_0008103 using a One Step Cloning Kit (ClonExpress II One Step Cloning Kit, Novozymes, China). The plasmids were constructed by cloning the circ_0061407 and circ_0008103 fragments into the pEnCMV-1-3xFLAG-SV40-Neo vector (Miaoling Biology, China) using specific primers. The recombinant plasmids were named pEnCMV-1-3xFLAG-SV40-Neo-circ_0008103 and pEnCMV-1-3xFLAG-SV40-Neo-circ_0061407. The circRNA overexpression plasmids were tested using DNA sequencing (performed by the Beijing Institute for Genome Research, China).

### Primer design

As previously described [17, 24, 25], circRNA primers for qPCR were designed using Primer 3 Plus (https://www.primer3plus.com/index.html) [26]. All primers were synthesized by BGI (Beijing Genomics Institution, China). The primer sequences used in this study are listed in Additional file 5: Table S1.

### RNA extraction, cDNA synthesis, and qPCR

Total RNA was extracted from cells and exosomes using TRIzol reagent (Invitrogen, USA) following a previously described protocol [24, 25]. cDNA was synthesized using ReverTra Ace qPCR RT Master Mix with gDNA Remover (TOYOBO, Japan) according to the manufacturer’s instructions. Each qPCR reaction system (10 μL) included 5 μL of Hieff® qPCR SYBR Green Master Mix (Yeasen Biotech, China), 3 μL of RNase-free water, 0.5 μL of forward primer (10 μmol/L), 0.5 μL of reverse primer (10 μmol/L), and 1 μL of cDNA solution. The qPCR was conducted on the ABI 7500 real-time PCR system (Applied Biosystems, USA) under the conditions as follows: denaturation at 95 °C for 5 min, 40 cycles at 94 °C for 30 s, 60 °C for 30 s, and 72 °C for 30 s. Finally, the reaction was held at 98 °C for 10 min, followed by a final hold at 4 °C. Each reaction was run in triplicate.

In the present study, GAPDH was chosen as the internal reference gene for circRNAs to normalize the qPCR data according to previous publications [27, 28]. In the subsets of healthy, pneumonia, benign lung tumours, and NSCLC, we calculated mean GAPDH Cq values of 25.06 (SD = 1.06), 24.17 (SD = 0.71), 24.95 (SD = 0.87), and 25.38 (SD = 0.94), respectively. The relative levels of circRNAs were calculated using the 2^−ΔCq^ method, where ΔCq was calculated by subtracting the average Cq values of GAPDH from the average Cq value of circRNAs.

### Western blotting

Cells or exosomes were lysed using radioimmunoprecipitation assay buffer supplemented with 1-mM phenylmethylsulfonyl fluoride and phosphatase inhibitors (Beyotime, China) as described previously [27]. The concentrations of extracted proteins were determined using a BCA protein assay kit (Beyotime, China) according to the manufacturer’s instructions. The extracted proteins were added to 8% polyacrylamide gel and separated through electrophoresis. The separated proteins were transferred to PVDF membranes (Millipore, USA). Following the incubation of the PVDF membranes with 5% skimmed milk for 1 h at room temperature, the PVDF membranes were incubated with primary antibodies overnight at 4 °C. Then, the membranes were washed using TBST buffer and incubated with secondary antibodies for 1 h at room temperature. After washing the membrane with TBST buffer, the protein bands on the PVDF membranes were detected with enhanced chemiluminescence (ECL) reagent (Advansta, USA) under an infrared imaging system (Li-COR, USA). The primary and secondary antibodies used for western blotting are listed in Additional file 6: Table S2.

### CircRNA microarray analysis

Exosomes from the serum of four healthy individuals and four patients with distant metastatic NSCLC were precipitated using the Serum Total Exosome Isolation Kit (Life Technologies, USA) and RNAs were extracted using TRIzol reagent (Invitrogen, USA). The circRNA profile was analyzed by the Arraystar Human circRNA Array (Arraystar, Rockville, MD, USA) according to the Arraystar Super RNA Labeling protocol. The screening criteria for differentially expressed circRNAs between the two groups of healthy and distant metastatic NSCLC were by a log2 fold change of > 7.5 and P-values of < 0.001.

### Agarose gel electrophoresis

We prepared a 2% agarose gel based on the size of the qPCR products of circRNAs. The circRNA fragments run at 120 V for 40 min and the bands were visualized and photographed using Gel Doc™ XR + (BIO-RAD, USA).

### Cell proliferation assay

The short-term cell viability was determined using the CCK-8 assay. After 48 h of treatment, 2000 cells were placed in 96-well plates (Jet Biofil, China). Next, 10 μL of CCK-8 reagent (APExBIO, USA) was added to the wells and incubated for 2 h. The absorbance was measured at 450 nm by a microplate absorbance reader (iMark, BIO-RAD, USA). To evaluate the long-term cell viability, 2000 cells were placed in 6-well plates for clone formation assay. After incubation for 14 days, cells were stained with 0.1% crystal violet staining solution (Solarbio, China). Colonies were counted using ImageJ software (version 1.8.0, USA). These experiments were performed in triplicate.

### Wound healing assay

The transfected cells were seeded in 6-well plates. Once the cells reached 95% confluence, the cell monolayer was scratched using a sterile 200 µL pipette tip. The cells were then incubated with a culture medium containing 2% FBS. The migrated cells at the wound were observed at 0 h, 24 h, and 48 h using a microscope (Olympus, Japan). The cells were washed with phosphate buffer solution (PBS) before photography. The migration distance of cells was measured using Image-Pro Plus 6.0 software. The experiment was repeated three times.

### Transwell migration and invasion assays

The transwell migration and invasion assays were conducted to evaluate the migration and invasive abilities of cells. The transwell chambers (8-μm pore size; Corning Incorporated, USA) coated without or with adhesive matrix (BD Biosciences Incorporated, USA) were used for migration and invasion assays, respectively. In the upper chamber, 20, 000 transfected cells resuspended in 200 μL of FBS-free medium were seeded, and 700 μL of medium containing 10% FBS was added to the lower chamber. After incubation at 37 °C for 24 h, the upper chamber was washed with PBS and then stained with 0.1% crystal violet staining solution (Solarbio, China). The cells were photographed using a microscope (Olympus, Japan) and counted using ImageJ software (version 1.8.0, USA). We repeated each experiment three times.

### Confocal microscopy

We used 1,1’-dioctadecyl-3,3,3’,3’-tetramethylindocarbocyanine perchlorate (Dil; Sigma, USA) to label exosomes. The nucleus was stained using DAPI (Solarbio, China). The exosomes extracted from the cell culture medium were resuspended and mixed with Dil dye at a ratio of 1:100. After incubation for 30 min in the dark at room temperature, the stained exosomes were co-incubated with the recipient cells for 12 h. Next, the cells were fixed using 4% paraformaldehyde (Solarbio, China) for 10 min at 4 °C. The exosome uptake by receptor cells was visualized using a confocal microscope (LEICA TCS SP8, Germany).

### Isolation and characterization of exosomes

Exosomes secreted by cells were extracted in this study by differential ultracentrifugation. Briefly, a four-step sequential ultracentrifugation was performed at 4 °C: centrifugation at 300 × g for 15 min to remove cells; centrifugation at 10,000 × g for 30 min to remove cellular debris; ultracentrifugation at 110,000 × g for 70 min to precipitate the exosomes, which were then resuspended in PBS and filtered through a membrane filter (0.22 μm in diameter); and finally, centrifugation at 110,000 × g for 70 min to remove soluble proteins. The exosomes were then resuspended in PBS and filtered through a membrane filter (0.22 μm in diameter). Serum exosomes were isolated by the Serum Total Exosome Isolation Kit (Life Technologies, USA) according to the manufacturer’s instructions. We performed western blotting to detect exosome-specific proteins (CD63, CD9, TSG, and CD81) and exosome-negative markers (calnexin) as previously described [29]. We further observed the morphology characteristics of exosomes by transmission electron microscope (HITACHI, Japan); and used a potentiometric analyzer (Zetasizer, ZEN3700, Malvern, UK) to detect the size of exosomes.

### Bioinformatic analysis

CircBase (http://circbase.org/) was used to reveal the sequence and genomic location of circ_0061407 and circ_0008103 [30]. CircBank (http://www.circbank.cn/) [31] and circAtlas (circatlas.biols.ac.cn) databases [32] were utilized to identify miRNAs that interact with circ_0061407 and circ_0008103 and to predict the methylation of the circRNAs. Additionally, we used the Cancer-Specific CircRNA (CSCD, http://gb.whu.edu.cn/CSCD/#) to predict the RNA-binding proteins and the protein coding capacity of circRNAs [33]. The databases, including miRTarBase (mirtarbase.cuhk.edu.cn) [34], miRDB (http://www.mirdb.org/) [35], and TargetScan (targetscan.org/vert_80/) [36] were employed to predict the target genes of miRNAs. To examine the expression levels of the target genes in NSCLC, we extracted a lung cancer dataset from The Cancer Genome Atlas (TCGA) database using the R software (version 4.2.1, USA). Gene Ontology (GO) and Kyoto Encyclopedia of Genes and Genomes (KEGG) pathway enrichment analyses were conducted with a bioinformatics web (https://www.bioinformatics.com.cn) [37].

### Establishment of tumour xenograft models

A total of 15 female BALB/c nude mice (age, 3 weeks) were purchased from Shanghai Slaccas Laboratory Animal Co., Ltd. The mice were randomly divided into 3 groups (5 mice/group). To examine the influence of circ_0061407 and circ_0008103 on tumour growth, stable SPC-A-1 cells overexpressing circ_0061407 (pEnCMV-1-3xFLAG-SV40-Neo-circ_0061407) and circ_0008103 (pEnCMV-1-3xFLAG-SV40-Neo-circ_0008103) were constructed. A total of 1 × 10^7^ SPC-A-1 cells were injected under the axillary skin of nude mice. Three weeks later, the mice were sacrificed and tumours were subsequently harvested. The maximum length (L), minimum length (W), and weight of tumours were measured. Tumour volume was calculated as ½LW^2^. All experiments involving animals were approved by the Experimental Animal Ethics Committee of the Health Science Center of Ningbo University.

### Statistical analysis

The statistical analyses were carried out with the SPSS 26.0 software (Chicago, USA) and GraphPad Prism 8.0 (GraphPad Software, USA). For in vitro experiments, we used the t-test to compare the data between the two groups. The two-way ANOVA was utilized to compare the data about CCK-8 assays. After converting the relative levels of circRNAs into normal distribution by LN, we compared the levels of circRNAs among the cohorts of healthy, pneumonia, benign lung tumours, and NSCLC using ANOVA and Tukey’s HSD test. To compare the levels of circRNAs between two subgroups, including M0 and M1, N0 and N1-3, tumour sizes of ≤ 5 cm vs. > 5 cm, stages I-II and III-IV, we used the nonparametric Mann–Whitney *U* test. The diagnostic ability of circRNAs was determined using receiver operating characteristic (ROC) curves, and the area under the curve (AUC) was calculated. The sensitivity and specificity were calculated when the Youden index (sensitivity + specificity—1) was maximum. A P-value of < 0.05 was considered statistically significantly different, and all P-values were two-sided.

## Results

### Profiling of circRNAs present in the serum exosomes of patients with distant metastatic NSCLC and characterization of circ_0061407 and circ_0008103

**Fig. 1:**
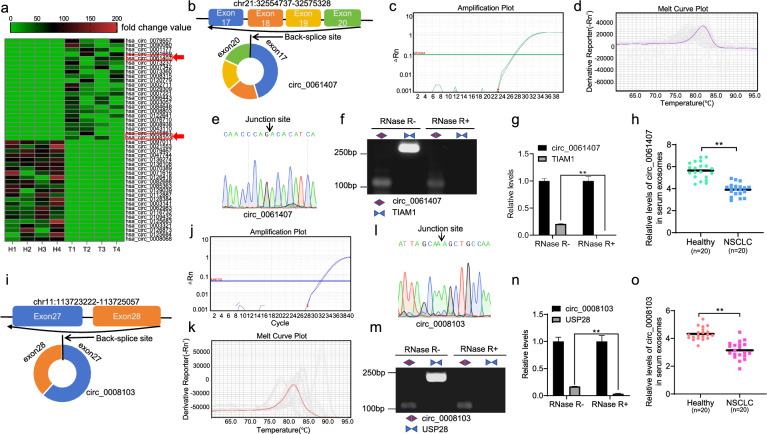
The screening of NSCLC-related circ_0008103 and circ_0061407 in serum exosomes. **a** Heatmap shows the top 46 differentially expressed (biggest log_2_ fold change values with smallest P-values) circRNAs in the exosomes from patients with NSCLC (n = 4), compared to healthy individuals (n = 4). The different numbers with colored bars represent the fold change values. **b** Schematic illustration of the circularization of circ_0061407. **c** Amplification curves of circ_0061407 from qPCR. **d** Melting curves of circ_0061407 from qPCR.** e** The back-splicing junction site of circ_0061407 was verified using Sanger sequencing. **f** The qPCR products of circ_0061407 and linear TIAM1 were tested by agarose gel electrophoresis. **g** Relative levels of the linear TIAM1 transcript and circ_0061407 after RNase R digestion. **h** The dot plot shows the relative levels of serum exosomal circ_0061407 in the healthy subset (n = 20) and the NSCLC subset (n = 20). **i** Schematic illustration of the circularization of circ_0008103. **j** Amplification curves of circ_0008103 from qPCR. **k** Melting curves of circ_0008103 from qPCR.** l** The back-splicing junction site of circ_0008103 was verified using Sanger sequencing. **m** The qPCR products of circ_0008103 and linear USP28 were tested by agarose gel electrophoresis. **n** Relative levels of the linear USP28 transcript and circ_0008103 after RNase R digestion. **o** The dot plot shows the relative levels of serum exosomal circ_0008103 in the healthy subset (n = 20) and the NSCLC subset (n = 20). **P < 0.01

To explore the expression pattern of circRNAs in the serum exosomes of patients with distant metastatic NSCLC, we extracted RNAs from exosomes in the serum of 4 healthy individuals and 4 patients with distant metastatic NSCLC and performed circRNA microarray analysis. According to the screening criteria of P-values (< 0.001) and log_2_ fold change values (> 7.5), a total of 324 differentially expressed circRNAs between healthy individuals and patients with distant metastatic NSCLC were selected. The top 46 circRNAs with the biggest log_2_ fold change values and the smallest P-values were shown in Fig. 1a and Additional file 7: Table S3, of which 23 circRNAs in the exosomes from randomly selected serum samples were detected by qPCR. Circ_0061407 and circ_0008103 could be successfully amplified. Circ_0061407 is derived from the 17 to 20 exons of the T lymphoma invasion and metastasis 1 (TIAM1) parent gene, which is located on chromosome 21 (Fig. 1b). The TCGA cohort (TCGA-LUAD/LUSC dataset) shows much lower expression levels of the TIAM1 gene in lung cancer tissues than those in normal tissues (Additional file 1: Fig. S1a). Circ_0008103 is derived from the 27 and 28 exons of the ubiquitin specific peptidase 28 (USP28) gene, which is located on chromosome 11 (Fig. 1i). The expression levels of the USP28 gene in lung cancer tissues were higher compared to the normal tissues (Additional file 1: Fig. S1b). qPCR was performed to quantify circ_0061407 (Fig. 1c, d) and circ_0008103 (Fig. 1j, k), the amplification and the melt curves show a successful amplification. The back-splicing junction sites of circ_0061407 (Fig. 1e) and circ_0008103 (Fig. 1l) were verified using Sanger sequencing. To test the stability of circ_0061407 and circ_0008103, RNAs used for reverse transcription were pre-treated with RNase R. qPCR was conducted with the primers designed to amplify circ_0061407, linear TIAM1 transcript, circ_0008103, and linear USP28 transcript. It was found that circ_0061407 but not the linear TIAM1 transcript was resistant to RNase R digestion (Fig. 1f, g). Similarly, circ_0008103 but not the linear USP28 transcript was resistant to RNase R digestion (Fig. 1m, n). To preliminarily test the expression of exosomal circ_0061407 and circ_0008103 in NSCLC, we randomly selected the serum samples from 20 healthy individuals and 20 patients with NSCLC. The qPCR results revealed that the relative levels of circ_0061407 (Fig. 1h) and circ_0008103 (Fig. 1o) in serum exosomes were significantly lower in patients with NSCLC compared to healthy individuals. Taken together, we identified two significantly deregulated circRNAs (circ_0061407, circ_0008103) in the serum exosomes of NSCLC.

### The characterization of serum exosomes and the levels of cellular and exosomal circ_0061407 and circ_0008103

**Fig. 2:**
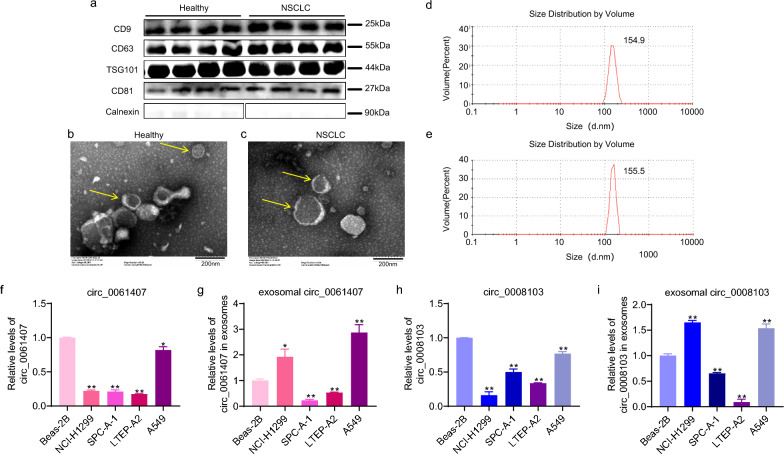
Characterization of serum exosomes and the quantification of cellular and exosomal circ_0061407 and circ_0008103.** a** Exosomal markers (CD9, CD63, TSG101, and CD81) and exosomal negative marker (Calnexin) were detected using Western blotting. **b**, **c** The typical cup-shaped morphology of the serum exosomes from healthy individuals and patients with NSCLC was characterized by transmission electron microscopy. **d**, **e** The average diameter of serum exosomes from healthy individuals and patients with NSCLC was detected using a potentiometric analyzer. **f** The levels of circ_0061407 in Beas-2B cells and lung cancer cells. **g** The levels of circ_0061407 in exosomes secreted by Beas-2B cells and lung cancer cells. **h** The levels of circ_0008103 in Beas-2B cells and lung cancer cells. **i** The levels of circ_0008103 in exosomes secreted by Beas-2B cells and lung cancer cells. *P < 0.05, **P < 0.01

In the present study, serum exosomes were precipitated by a serum exosome isolation kit. Western blotting was performed to detect the classical exosome proteins like CD63, CD9, CD81, and TSG101 and a negative protein calnexin (Fig. 2a). The typical cup-shaped morphology of the serum exosomes from healthy individuals (Fig. 2b) and patients with NSCLC (Fig. 2c) were characterized by a transmission electron microscopy. In addition, the size of serum exosomes was measured by a potentiometric analyzer. The average diameter of serum exosomes was 154.9 nm and 155.5 nm in healthy people (Fig. 2d) and patients with NSCLC (Fig. 2e), respectively. The presence of specific exosome proteins, the typical shape, and the size of precipitated exosomes suggest the successful isolation of exosomes from the serum of healthy individuals and patients with NSCLC. Following the selection of NSCLC-associated circ_0061407 and circ_0008103, we utilized qPCR to analyze their levels in lung cancer cell lines (NCI-H1299, A549, SPC-A-1, LTEP-A2), normal lung epithelial cells (Beas-2B), and in the exosomes secreted by the corresponding cells. We found that the levels of cellular circ_0061407 (Fig. 2f) and circ_0008103 (Fig. 2h) were decreased in lung cancer cells than those in Beas-2B cells. However, the levels of exosomal circ_0061407 and circ_0008103 exhibited variations in different lung cancer cell lines. Circ_0061407 (Fig. 2g) and circ_0008103 (Fig. 2i) were increased in the exosomes secreted by NCI-H1299, and A549 cells, while they were decreased in the exosomes from SPC-A-1, and LTEP-A2 cells compared to Beas-2B cells. This phenomenon indicates the involvement of circ_0061407 and circ_0008103 in NSCLC and the selective packaging of circ_0061407 and circ_0008103 into the exosomes secreted by different NSCLC cell lines. We chose SPC-A-1, and LTEP-A2 cells to further study the effect of circ_0061407 and circ_0008103 overexpression on cell proliferation, migration, and invasive abilities.

### The influence of circ_0061407 and circ_0008103 overexpression on NSCLC in vitro and in vivo

**Fig. 3:**
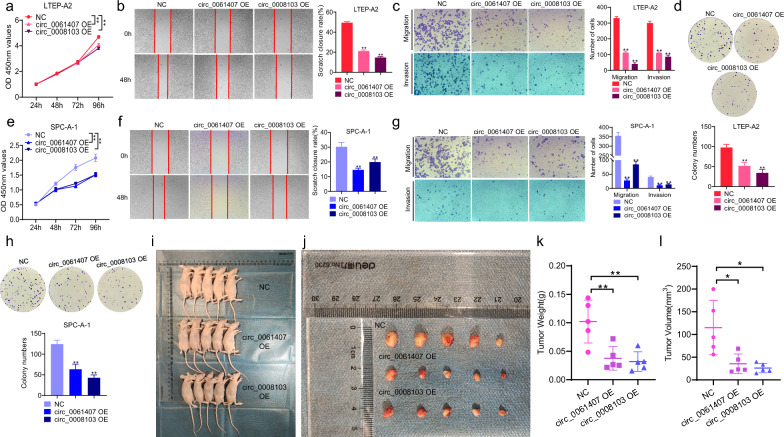
Circ_0061407 and circ_0008103 inhibit NSCLC cell proliferation, migration, and invasion. **a**, **e** CCK-8 assay was used to determine the effect of upregulated circ_0061407 and circ_0008103 on the short-term proliferation of LTEP-A2 and SPC-A-1 cells. **b**, **f** Wound healing assay was performed to detect the effect of circ_0061407 and circ_0008103 overexpression on the migration ability of LTEP-A2 and SPC-A-1 cells. **c**, **g** Transwell migration and invasion assays were conducted to evaluate the impact of upregulated circ_0061407 and circ_0008103 on the migration and invasion abilities of LTEP-A2 and SPC-A-1 cells. **d, h** Clone formation assay was performed to detect the effect of circ_0061407 and circ_0008103 overexpression on the long-term proliferative capacity of LTEP-A2 and SPC-A-1 cells. **i**, **j** The nude mice were subcutaneously injected with the stable SPC-A-1 cells overexpressing circ_0061407 and circ_0008103, and the tumours were collected. **k**, **l** The box plots show the tumour weight and volume. *P < 0.05, **P < 0.01

To investigate the role of circ_00061407 and circ_0008103 in NSCLC, we constructed overexpression plasmids of circ_0061407 and circ_0008103 to upregulate their levels. qPCR analyses show the obvious upregulation of circ_0061407 (Additional file 2: Fig. S2a) and circ_0008103 (Additional file 2: Fig. S2b) after the transfection of overexpression plasmids to Beas-2B and lung cancer cells. Next, we performed CCK-8, wound healing, transwell, and colony formation assays to study the effect of circ_0061407 and circ_0008103 overexpression. It was found that upregulated circ_0061407 and circ_0008103 inhibited the short-term and long-term proliferation of LTEP-A2 (Fig. 3a, d) and SPC-A-1 (Fig. 3e, h) cells. Additionally, the overexpression of circ_0061407 and circ_0008103 was able to inhibit the migration and invasive abilities of LTEP-A2 (Fig. 3b, c) and SPC-A-1 (Fig. 3f, g) cells. To further investigate the role of circ_0061407 and circ_0008103 in tumour growth, we established a subcutaneous xenograft tumour model using stable SPC-A-1 cells overexpressing circ_0061407 and circ_0008103. We found decreased tumour weight and volume in the circ_0061407 and circ_0008103 overexpression group compared to the control group (Fig. 3i-l). Overall, these results revealed that circ_0061407 and circ_0008103 might repress the proliferation, migration, invasion capabilities of NSCLC cells, and the lung tumour growth.

### Transport of circ_0008103 and circ_0061407 by exosomes

**Fig. 4:**
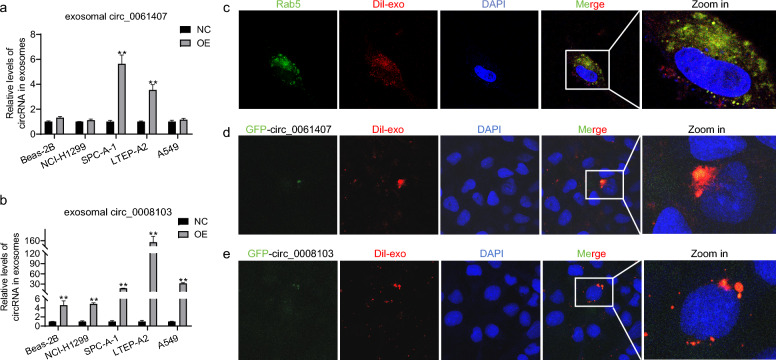
The transport of circ_0061407 and circ_0008103 by exosomes. **a**, **b** The levels of circ_0061407 and circ_0008103 in exosomes form Beas-2B and lung cancer cells transfected with circ_0008103 and circ_0061407 overexpression plasmids. **c** The co-localization of exosomes with endogenous Rab5 in the cytoplasm of Beas-2B cells. **d, e** Circ_0061407 and circ_0008103 within the exosomes from transfected LTEP-A2 cells entered the cytoplasm of Beas-2B cells. **P < 0.01

Considering the suppressive role of circ_0008103 and circ_0061407 in NSCLC cells’ migration and invasion, and the down-regulation of circ_0008103 and circ_0061407 in the exosomes from lung cancer cells, we were interested in whether circ_0008103 and circ_0061407 could affect the oncogenic behavior of recipient cells by the transport of exosomes. To answer this question, we initially delivered the circ_0008103 and circ_0061407 overexpression plasmids into Beas-2B and lung cancer cells. We collected the exosomes secreted by the cells and quantified the levels of circ_0008103 and circ_0061407 in the exosomes. The qPCR results show a significant increase of circ_0061407 (Fig. 4a) and circ_0008103 (Fig. 4b) levels in the exosomes secreted particularly by the SPC-A-1 and LTEP-A2 cells. Previous studies have reported that Rab5 protein plays a crucial role in exosome biogenesis [38]. Therefore, we transfected Beas-2B cells with a GFP-tagged Rab5 plasmid and further co-cultured the transfected Beas-2B cells with the exosomes secreted by LTEP-A2 cells. We observed a co-localization of exosomes with endogenous Rab5 in the cytoplasm of Beas-2B cells (Fig. 4c), suggesting LTEP-A2 derived exosomes could enter into Beas-2B cells. We further transfected LTEP-A2 cells with the circ_0008103 and circ_0061407 overexpression plasmids tagged by GFP and collected exosomes secreted by the transfected LTEP-A2 cells. Then, we co-cultured the isolated exosomes (labeled by Dil) with Beas-2B cells. We observed a co-localization of circ_0061407 (Fig. 4d) and circ_0008103 (Fig. 4e) with the exosomes from LTEP-A2 cells in the cytoplasm of Beas-2B cells. These results suggest that the exosomes from LTEP-A2 cells could encapsulate circ_0061407 and circ_0008103 and transport them into Beas-2B cells.

### Exosome-transported circ_0061407 and circ_0008103 inhibit recipient cells’ proliferation, migration, and invasion

**Fig. 5:**
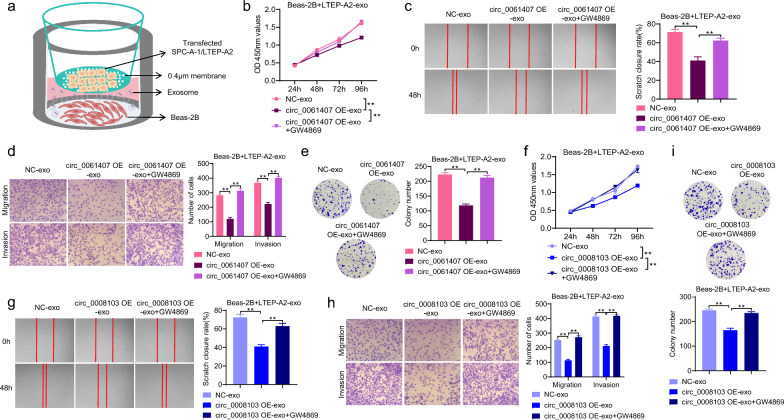
The role of exosome-transported circ_0061407 and circ_0008103 in recipient cells. **a** A co-culture device was used to study the effect of exosomes secreted by transfected LTEP-A2 and SPC-A-1 cells on Beas-2B cells. **b**, **f** The short-term proliferative capacity of Beas-2B cells was evaluated using the CCK-8 assay. **c**, **g** Wound healing assay was used to assess the migratory capacity of Beas-2B cells. **d**, **h** Transwell assay was used to evaluate the migration and invasion abilities of Beas-2B cells. **e**, **i** Colony formation assay was used to detect the clone formation ability of Beas-2B cells. **P < 0.01

To investigate the potential impact of circ_0008103 and circ_0061407 after the transport by exosomes on the recipient cells, we assembled a co-culture device as shown in Fig. 5a. The co-culture device comprises the upper and lower chambers separated by a membrane with 0.4 µm pores, which allows exosomes released by transfected LTEP-A2 or SPC-A-1 cells to enter the lower chamber. Specifically, LTEP-A2 and SPC-A-1 cells were transfected with circ_0008103 and circ_0061407 overexpression plasmids, and placed into the upper chamber. Following the co-culture of Beas-2B cells in the lower chamber with the exosomes secreted by transfected LTEP-A2 or SPC-A-1 cells, the viability, migration, and invasive abilities of Beas-2B cells were tested. It was found that the exosomes released by LTEP-A2 transfected with circ_0061407 (Fig. 5b, e) and circ_0008103 (Fig. 5f, i) overexpression plasmids inhibit the short-term and long-term proliferation of Beas-2B cells. Additionally, the migration and invasive abilities of Beas-2B cells were weakened after the incubation with the exosomes from LTEP-A2 cells transfected with circ_0061407 (Fig. 5c, d) and circ_0008103 (Fig. 5g, h) overexpression plasmids. Additionally, to further investigate the role of circ_0008103 and circ_0061407 transported by exosomes in NSCLC, we inhibited exosome release with the exosome secretion inhibitor GW4869 (10 μM) to recipient cells. Delivery of circ_0008103 and circ_0061407 via exosomes from LTEP-A2 cells inhibited proliferation, migration, and invasion of Beas-2B cells, however, the usage of GW4869 attenuated the effects (Fig. 5b-i). Besides LTEP-A2 cells, SPC-A-1 cells were also transfected with circ_0008103 and circ_0061407 overexpression plasmids and seeded to the upper chamber for the co-culture with Beas-2B cells. The exosomes released from the transfected SPC-A-1 cells showed a similar inhibitory effect on the proliferation, migration, and invasion of Beas-2B cells (Additional file 3: Fig. S3) to the exosomes from transfected LTEP-A2 cells (Fig. 5). Taken together, these results indicate that circ_0008103 and circ_0061407 delivered by exosomes might prevent cell proliferation, migration, and invasion of recipient cells.

### Diagnostic potential of exosomal circ_0008103 and circ_0061407 for NSCLC

**Fig. 6:**
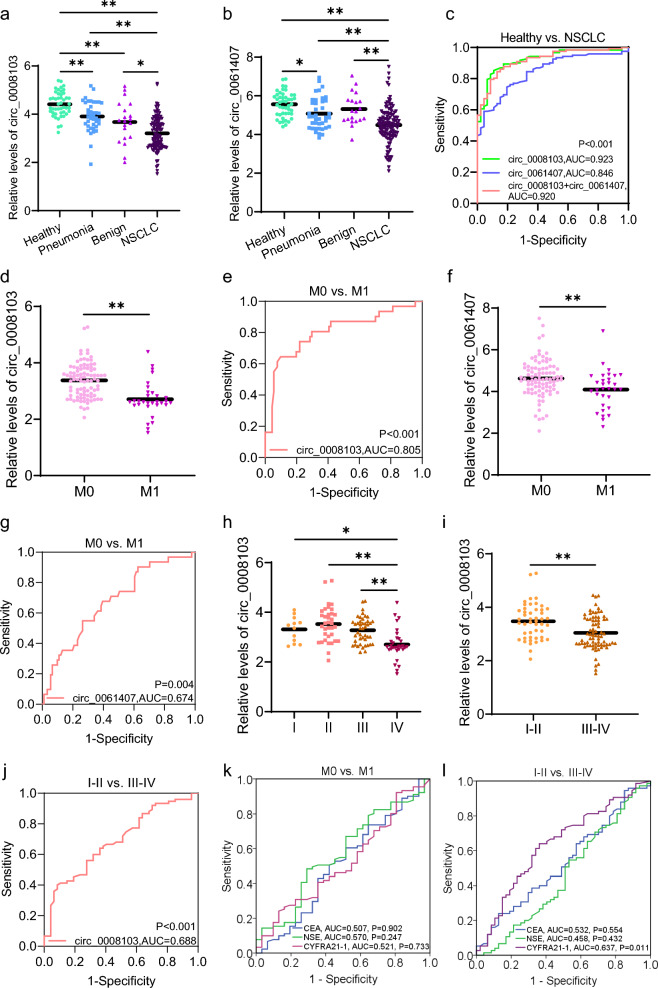
Diagnostic potential of serum exosomal circ_0008103 and circ_0061407 for NSCLC. **a**, **b** The box plots show the levels of serum exosomal circ_0008103 and circ_0061407 in four cohorts: healthy (n = 46), pneumonia (n = 40), benign lung tumours (n = 20), and NSCLC (n = 122). The relative levels of serum exosomal circ_0008103 and circ_0061407 were transformed by LN to obtain normal distribution, and compared using ANOVA and Tukey’s HSD test. **c** ROC analyses were performed to test the diagnostic power of serum exosomal circ_0008103 and circ_0061407, and their combination to discriminate healthy from NSCLC. **d**, **h**, **i** The box plots show the levels of serum exosomal circ_0008103 in the subgroups of without/with distant metastases (M0/M1) (n = 91/31), and tumour stages I/II/III/IV (n = 12/35/44/31), and tumour stages I-II (n = 47) and III-IV (n = 75). The levels of circ_0008103 between different subgroups were compared by nonparametric Mann–Whitney U test. **e**, **j** ROC analyses show the diagnostic power of serum exosomal levels of circ_0008103 to distinguish M0 and M1, as well as stages I-II and III-IV. **f** The box plots show the levels of serum exosomal circ_0061407 in the subgroups of M0 (n = 91) and M1 (n = 31). **g** ROC analyses show the diagnostic power of serum exosomal levels of circ_0061407 to distinguish M0 and M1. **k** ROC curves show the diagnostic value of CEA, NSE, and CYFRA21-1 to distinguish M0 and M1. **l** ROC curves show the potential of CEA, NSE, and CYFRA21-1 to discriminate stages I-II and III-IV NSCLC. *P < 0.05, **P < 0.01

Considering the tumour-suppressive effect of exosome-delivered circ_0008103 and circ_0061407 on recipient cells, we were interested in their relationship with NSCLC, and with the pathological parameters of NSCLC. We isolated exosomes from the serum of 122 patients with NSCLC, 20 patients with benign lung tumours, 40 patients with pneumonia, and 46 healthy individuals. We quantified the levels of exosomal circ_0008103 and circ_0061407 by qPCR and compared their levels among different cohorts. Exosomal circ_0008103 and circ_0061407 show similar expression patterns among the cohorts of NSCLC, benign lung tumours, pneumonia, and healthy. Overall, the levels of exosomal circ_0008103 (Fig. 6a) and circ_0061407 (Fig. 6b) gradually declined from healthy, pneumonia, benign lung tumours to NSCLC, and were significantly lower in the serum of patients with NSCLC compared to those in healthy individuals. ROC analyses show the AUC values, the sensitivity and specificity of circ_0008103 (0.923, 89.1% sensitivity, 86.1% specificity), circ_0061407 (0.846, 83.5% sensitivity, 85.2% specificity), and the combination of circ_0008103 and circ_0061407 (0.920, 84.4% sensitivity, 87.0% specificity) to distinguish healthy from NSCLC (Fig. 6c). Next, we evaluated the association of exosomal circ_0008103 and circ_0061407 with clinical parameters of NSCLC, including histological subtype, lymph node metastasis, distant metastasis, tumour stage, and tumour size. As shown in Additional file 8: Table S4, the levels of exosomal circ_0008103 and circ_0061407 had a strong correlation with distant metastasis but not with tumour size, tumour subtype, and lymph node metastasis. Besides, the levels of exosomal circ_0008103 were associated with the tumour stage. Specifically, the levels of circ_0008103 (Fig. 6d) and circ_0061407 (Fig. 6f) in the serum exosomes of the M1 subset were significantly lower than those in the M0 subgroup, with the AUC values of 0.805 Fig. 6e, 64.5% sensitivity, 90.1% specificity) and 0.674 (Fig. 6g, 54.8% sensitivity, 73.6% specificity), respectively, to discriminate the M0 and M1 subgroups. Regarding the tumour stage, it was found that the levels of exosomal circ_0008103 in advanced stage IV were lower than those in stages I, II, and III (Fig. 6h). And there was a statistically significant down-regulation of exosomal circ_0008103 in the subgroup of tumour stages III-IV compared to the tumour stages I-II (Fig. 6i), with an AUC value of 0.688 (40.0% sensitivity, 91.5% specificity) to differentiate stages I-II and III-IV (Fig. 6j). However, the levels of serum exosomal circ_0061407 show no significant difference among tumour stages I, II, III, and III (data not shown). Additionally, we collected the values of traditional lung tumour markers (CEA, NSE, and CYFRA21-1) from the patients with NSCLC and performed association analyses with clinical parameters. Among the three traditional lung tumour markers, only CYFRA21-1 was linked to lymph node metastasis and tumour stages (Additional file 8: Table S4). We further performed ROC analyses to compare their diagnostic abilities with exosomal circ_0008103 and circ_0061407 to discriminate M0 and M1, tumour stages I-II and III-IV subpopulations. It could be found that exosomal circ_0008103 had a higher AUC value (Fig. 6e, 0.805) than CEA (Fig. 6k, 0.507), NSE (Fig. 6k, 0.570), and CYFRA21-1 (Fig. 6k, 0.521) to distinguish M0 and M1 subsets, and a higher AUC value (Fig. 6j, 0.688) than CEA (Fig. 6l, 0.532), NSE (Fig. 6l, 0.458), and CYFRA21-1 (Fig. 6l, 0.637) to discriminate stages I-II and III-IV. For exosomal circ_0061407, it also shows a higher AUC value of 0.674 (Fig. 6g) than CEA, NSE, and CYFRA21-1 (Fig. 6k) to distinguish M0 and M1 subgroups. These findings imply that circ_0008103 and circ_0061407 present in serum exosomes were closely linked to NSCLC, and had correlations with the aggressive characteristics (distant metastasis, advanced tumour stages) of NSCLC, showing superior diagnostic power to discriminate M0 and M1, stages I-II and III-IV subpopulations than CEA, NSE, and CYFRA21-1.

### Functional mechanisms of circ_0061407 and circ_0008103 predicted by bioinformatic analyses

**Fig. 7:**
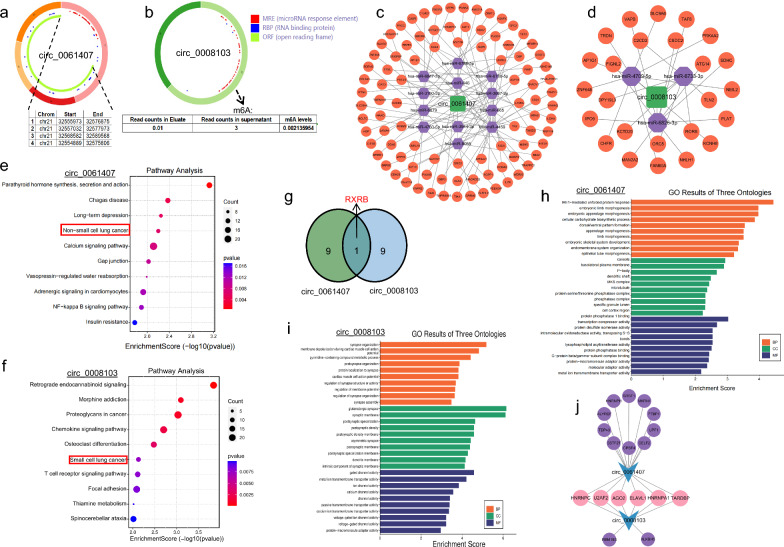
Functional mechanisms of circ_0061407 and circ_0008103 predicted by bioinformatic analyses. **a**, **b** Structural patterns of circ_0061407 and circ_0008103 predicted by the Cancer-Specific CircRNA (CSCD, http://gb.whu.edu.cn/CSCD/#). CSCD predicted the potential of circ_0061407 to encode polypeptides. The possibility of m6A modification of circ_0008103 was predicted by circbank (http://www.circbank.cn/searchCirc.html). **c**, **d** The circRNA-miRNA-mRNA regulatory network of circ_0061407 and circ_0008103 was constructed using the Cytoscape software. KEGG pathway analysis was performed with the circRNA-associated mRNAs predicted by TargetScan. **e** The involved pathways of mRNAs indirectly interacted with circ_0061407. **f** The involved pathways of mRNAs predicted by circ_0008103. **g** RXRB might interact with circ_0061407 and circ_0008103 in the lung cancer pathway. GO enrichment analyses were performed using the target mRNAs predicted by TargetScan. **h**, **i** The mRNAs related to circ_0061407 and circ_0008103 were categorized into three ontologies, including molecular function, cellular component, and biological process.** j** The potential RNA-binding proteins with circ_0061407 and circ_0008103 were predicted by CSCD

Given the tumour-suppressive role of circ_0061407 and circ_0008103 and their diagnostic value in NSCLC, we were inspired to explore their potential mechanisms by bioinformatic analyses. The CSCD (http://gb.whu.edu.cn/CSCD/#) and circbank (http://www.circbank.cn/searchCirc.html) databases revealed the basic structural patterns and diverse biological functions of circ_0061407 (Fig. 7a) and circ_0008103 (Fig. 7b), including their abilities to act as miRNA sponges, protein traps, and even templates for polypeptide synthesis. There is growing evidence that circRNAs may function as miRNA sponges to regulate tumourigenesis and development [39]. The potential interacted miRNAs with circ_0061407 and circ_0008103 were predicted by several bioinformatic databases, including Pita (http://genie.weizmann.ac.il/pubs/mir07/mir07data.html) [40], miRanda (http://www.microrna.org/microrna/home.do) [41], and Targetscan (http://www.targetscan.org/). It was found that circ_0061407 (Additional file 4: Fig. S4a) and circ_0008103 (Additional file 4: Fig. S4b) had 12 and 3 shared target miRNAs, respectively, by the above 3 databases. We further used these shared miRNAs to predict their target mRNAs using databases, like miRTarBase (mirtarbase.cuhk.edu.cn), miRDB (http://www.mirdb.org/), and TargetScan, and constructed the circRNA-miRNA-mRNA regulatory network for circ_0061407 (Fig. 7c) and circ_0008103 (Fig. 7d). To further explore the signaling pathways the circRNA-associated mRNAs involved in, we performed KEGG pathway analyses with the mRNAs predicted by TargetScan. It was uncovered that the mRNAs indirectly interacted with circ_0061407 were significantly enriched in the NSCLC pathway (Fig. 7e), whereas the mRNAs predicted by circ_0008103 were significantly enriched in small cell lung cancer (Fig. 7f), implying their contribution to lung cancer. The genes enriched in the NSCLC pathway and those enriched in the small cell lung cancer pathway were summarized in Additional file 9: Table S5, of which retinoid X receptor beta (RXRB) is associated with circ_0061407 and circ_0008103 (Fig. 7g). RXRB is a member of the retinoid X receptor families involved in cancer development and stemness [42]. We compared the expression levels of RXRB in the tissues of patients with lung cancer and healthy people with the TCGA cohort (TCGA-LUAD/LUSC dataset). We found that the expression levels of RXRB in the tissues of patients with lung cancer were higher than those in the tissues of healthy people (Additional file 4: Fig. S4c), suggesting its correlation with lung cancer. Additionally, GO enrichment analyses were performed using the target mRNAs predicted by TargetScan. The mRNAs related to circ_0061407 (Fig. 7h) and circ_0008103 (Fig. 7i) were categorized into three ontologies, including molecular function, cellular component, and biological process. The CSCD database (http://gb.whu.edu.cn/CSCD/#) was used to predict potential RNA-binding proteins for circ_0061407 and circ_0008103, and 6 proteins (HNRNPC, U2AF2, AGO2, ELAVL1, HNRNPA1, and TARDBP) were found to interact with both circRNAs (Fig. 7j). More additional functions of circ_0008103 and circ_0061407 were searched through circbank (http://www.circbank.cn/searchCirc.html). As shown in Fig. 7a, there were 4 sites for circ_0061407 to serve as potential templates for polypeptide synthesis. And circ_0008103 has the possibility of m6A modification (Fig. 7b). Collectively, these results indicate the involvement of circ_0008103 and circ_0061407 in lung cancer and the underlying mechanisms through which they might affect the development of this disease.

## Discussion

Exosomes play a vital role in intercellular communication through shuttling their bioactive components among cells. CircRNAs are reliably present and highly enriched in exosomes. So far, exoRBase 2.0 (http://www.exorbase.org/) identified 79, 084 circRNAs in extracellular vesicles from human body fluids [43]. The bilayer lipid membrane of exosomes provides a protective barrier for circRNAs from the degradation by nucleases, making circRNAs enclosed in exosomes have a unique advantage as tumour markers. Additionally, circRNAs transported by exosomes have been frequently reported to play tumour-suppressive or oncogenic roles in tumour development. For example, Wang and colleagues identified a novel circRNA called circLPAR1 in the exosomes of patients with colorectal cancer [44]. They demonstrated that circLPAR1 encapsulated in exosomes could be internalized by colorectal cancer cells, and further suppressed tumour growth by directly bound with eukaryotic translation initiation factor 3 subunit h (eIF3h), suppressing the methyltransferase-like 3 (METTL3)-eIF3h interaction and decreasing the translation of oncogene bromodomain containing 4 [44].

In the present study, we discovered two dysregulated circRNAs (circ_0061407 and circ_0008103) in the serum exosomes of patients with metastatic NSCLC using circRNA microarray analysis. We revealed that these two circRNAs could suppress the proliferation, migration, and invasive abilities of lung cancer cells (Fig. 3). Moreover, we found that exosomes carrying circ_0061407 and circ_0008103 could enter recipient cells (Fig. 4), where the circRNAs were released and affected the viability, migration, and invasion of recipient cells (Fig. 5, Additional file 3: Fig. S3). Furthermore, we utilized qPCR to quantify the levels of circ_0061407 and circ_0008103 within the serum exosomes of healthy individuals, patients with pneumonia, patients with benign lung tumours, and patients with NSCLC. It was found that patients with NSCLC had lower levels of exosomal circ_0061407 and circ_0008103 compared to healthy individuals, patients with pneumonia, and patients with benign lung tumours (Fig. 6). Additionally, lower levels of exosomal circ_0061407 and circ_0008103 were closely correlated with more aggressive features (distant metastasis or advanced tumour stages) of NSCLC, showing superior diagnostic sensitivity and specificity than traditional tumour markers (CEA, NSE, and CYFRA21-1) to discriminate M0 and M1, tumour stages I-II and III-IV subpopulations (Fig. 6). Based on the tumour-suppressive role of circ_0061407 and circ_0008103, and their potential value to diagnose NSCLC, we were inspired to explore their functional mechanisms in lung cancer using bioinformatic databases. The KEGG pathway analyses uncovered the involvement of circ_0061407 and circ_0008103 in NSCLC and small-cell lung cancer, respectively, through competing with tumour-associated genes for the binding sites of miRNAs or interaction with proteins (Fig. 7). Additionally, circ_0061407 had the potential to be translational template for polypeptide or protein synthesis, while circ_0008103 could be modified by methylation, which might affect the function of circRNAs.

To our best knowledge, circ_0061407 and circ_0008103 are novel circRNAs that have not been reported in cancers and other diseases. Circ_0061407 is spliced from the 17 to 20 exons of the TIAM1 transcript, which was frequently reported as a tumour-associated gene. TIAM1 was elevated in thyroid cancer, and its knockdown repressed thyroid cancer cell proliferation [45]. In NSCLC, TIAM1 promoted the migration and invasion of NSCLC cells through interacting with tripartite motif 28, a master regulator of gene expression in the nucleus [46]. The methylation of TIAM1 contributed to the metastasis of colon cancer and could be a predictive factor for colon cancer prognosis and a potential target for metastasis inhibition [47]. USP28 encodes a deubiquitinase that governs various biological processes, including cellular proliferation, DNA damage repair, apoptosis, and oncogenesis [48]. USP28 acts as an oncoprotein by stabilizing crucial oncoproteins in cancers, such as the transcription factor c-MYC [49]. Therefore, the inhibition of USP28 using USP28 small molecule inhibitors is effective in the treatment of cancers, particularly in squamous cell carcinoma [48]. The bioinformatic analyses also showed abnormal levels of TIAM1 and USP28 genes in the lung cancer tissues (Additional file 1: Fig. S1) in the present study. We believed that there might be a correlation between the functions of circRNAs and their origin genes. However, the mechanisms leading to the dysregulation of circ_0061407 and circ_0008103 in NSCLC, such as the regulators affecting the splicing of circRNAs, the interaction of circRNAs with their origin transcripts, and the selective packaging of circRNAs to exosomes needs to be deeply investigated.

Altogether, the present study elucidates the tumour-repressive role of exosome-transported circ_0061407 and circ_0008103 and highlights their diagnostic value in NSCLC. However, further studies are needed to investigate the selective encapsulation of circ_0061407 and circ_0008103 into exosomes during the development of NSCLC, and the specific mechanisms through which circ_0061407 and circ_0008103 in exosomes participated in the progression of NSCLC in vitro and in vivo. In addition, a larger cohort of patients with NSCLC with a long-term follow-up should be recruited to verify the diagnostic value of circ_0061407 and circ_0008103.

## Conclusions

Collectively, our study identified two novel circRNAs (circ_0061407 and circ_0008103) associated with NSCLC in serum exosomes. We observed that circ_0061407 and circ_0008103 could be packaged into exosomes and transported to recipient cells, consequently inhibiting the viability, migration, and invasion of recipient cells. Besides, exosomal circ_0061407 and circ_0008103 showed dysregulated levels in patients with NSCLC and might discriminate patients with distant metastasis from those without distant metastasis. The study provides alternative biomolecules (circ_0061407 and circ_0008103) for targeted therapy and diagnosis of lung cancer.

### Supplementary Information

**Additional file 1: Fig. S1** The levels of the TIAM1 gene and USP28 gene in lung cancer. **a, b** TCGA cohort (TCGA-LUAD/LUSC dataset) shows the levels of TIAM1 and USP28 in normal (n = 108) and lung tumour (n = 1041) tissues. **P < 0.01.

**Additional file 2: Fig. S2** The transfection efficiency of circRNA overexpression plasmids. **a, b** The transfection efficiency of circ_0061407 and circ_0008103 overexpression plasmids in Beas-2B and lung cancer cells. **P < 0.01.

**Additional file 3: Fig. S3** The effect of exosome-transported circ_0061407 and circ_0008103 on recipient cells. **a, e** The proliferative capacity of Beas-2B cells was evaluated using the CCK-8 assay. **b, f** Wound healing assay was used to assess the migratory capacity of Beas-2B cells. **c, g** Transwell assay was performed to evaluate the migration and invasion abilities of Beas-2B cells. **d, h** Colony formation assay was used to detect the clone formation ability of Beas-2B cells. **P < 0.01.

**Additional file 4: Fig. S4** MiRNAs interacted with circ_0061407 and circ_0008103. **a, b** Using the Pita (http://genie.weizmann.ac.il/pubs/mir07/mir07_data.html), miRanda (http://www.microrna.org/microrna/home.do), and Targetscan databases (http://www.targetscan.org/), a Venn diagram was created to show the numbers of the miRNAs that were predicted to interact with circ_0061407 and circ_0008103. **c** TCGA cohort (TCGA-LUAD/LUSC dataset) shows the levels of RXRB in normal (n = 108) and lung tumour (n = 1041) tissues. **P < 0.01.

**Additional file 5: Table S1.** The sequences of primers used in the present study.

**Additional file 6: Table S2.** Antibodies used for western blotting.

**Additional file 7: Table S3.** The top 46 circRNAs with the biggest log_2_ fold change values and the smallest P-values in circRNA microarray analysis.

**Additional file 8: Table S4.** The comparison of the relative levels of serum exosomal circ_0008103, circ_0061407, and traditional tumour markers with clinical parameters of NSCLC by Mann–Whitney U test

**Additional file 9: Table S5. **The genes enriched in the NSCLC pathway (related to circ_0061407) and those enriched in the small cell lung cancer pathway (related to circ_0008103)

## Data Availability

The datasets used and/or analyzed during the current study are available from the corresponding author upon reasonable request.

## Abbreviations

CircRNA: Circular RNA
NSCLC: Non-small-cell lung cancer
MiRNA: MicroRNA
LncRNA: Long non-coding RNA
mRNA: Messenger RNA
EMT: Epithelial-mesenchymal transition
ncRNAs: Non-coding RNAs
qPCR: Quantitative polymerase chain reaction
ECL: Enhanced chemiluminescence
PBS: Phosphate buffer solution
ROC: Receiver operating characteristic
AUC: Area under the curve
TIAM1: T lymphoma invasion and metastasis 1
USP28: Ubiquitin specific peptidase 28
CEA: Carcinoembryonic antigen
NSE: Neuron-specific enolase
CYFRA21-1: Cytokeratin 19 fragment
KEGG: Kyoto Encyclopedia of Genes and Genomes
GO: Gene ontology
RXRB: Retinoid X receptor beta
METTL3: Methyltransferase-like 3
eIF3h: Eukaryotic translation initiation factor 3 subunit h
CeRNA: Competitive endogenous RNA
TCGA: The Cancer Genome Atlas
