# Supplementary figures and images for: Exosome-transported circ_0061407 and circ_0008103 play a tumour-repressive role and show diagnostic value in non-small-cell lung cancer
Source: J Transl Med. 2024 May 6;22:427. doi: 10.1186/s12967-024-05215-6 (PMC11071259; doi:10.1186/s12967-024-05215-6)

## Slide 1
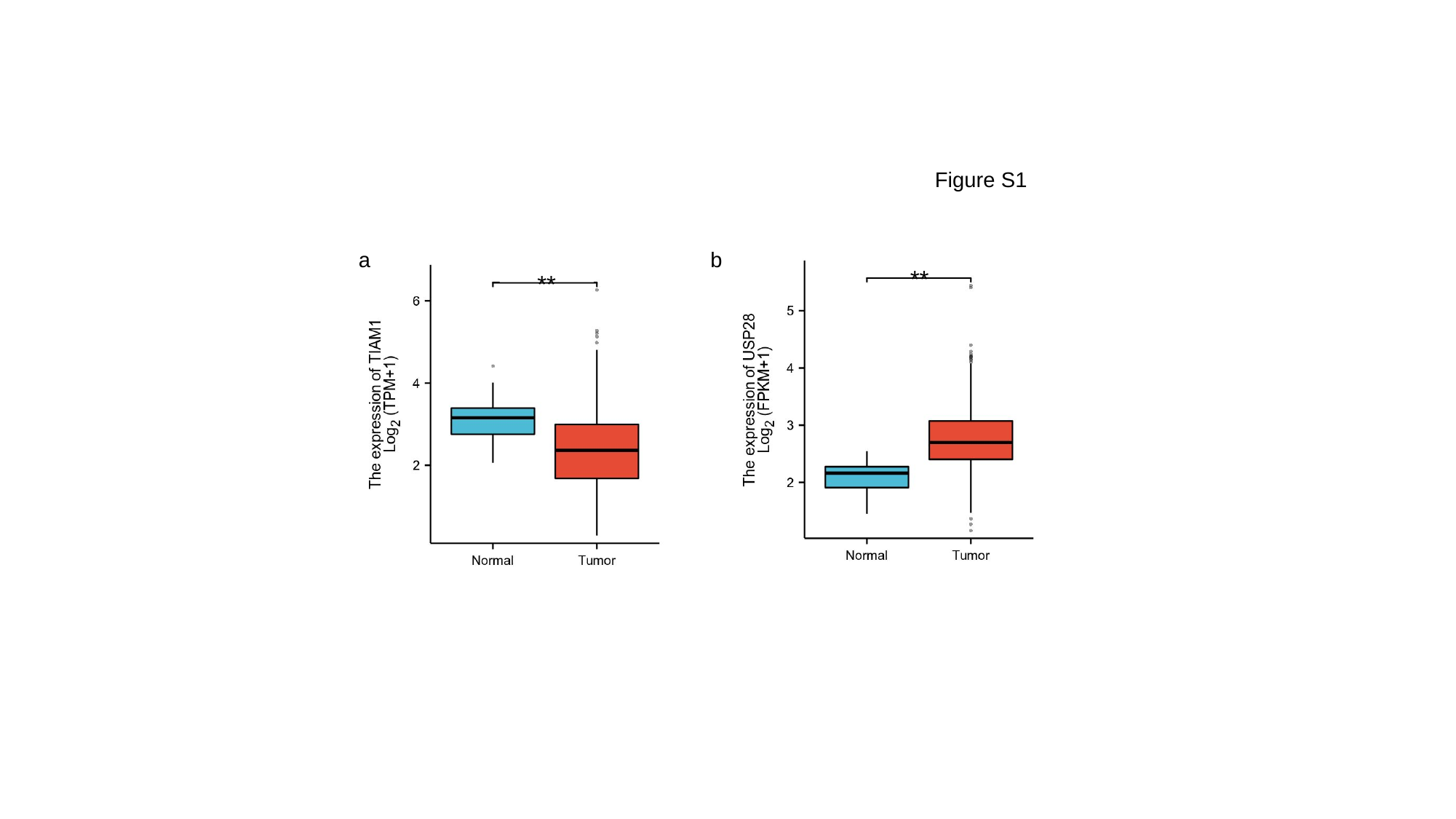

Figure S1
a
**
b
**

Supplement: Supplementary file 1 — Additional file 1: Fig. S1 The levels of the TIAM1 gene and USP28 gene in lung cancer. a, b TCGA cohort (TCGA-LUAD/LUSC dataset) shows the levels of TIAM1 and USP28 in normal (n = 108) and lung tumour (n = 1041) tissues. **P < 0.01. [file 12967_2024_5215_MOESM1_ESM.pptx]

## Slide 1
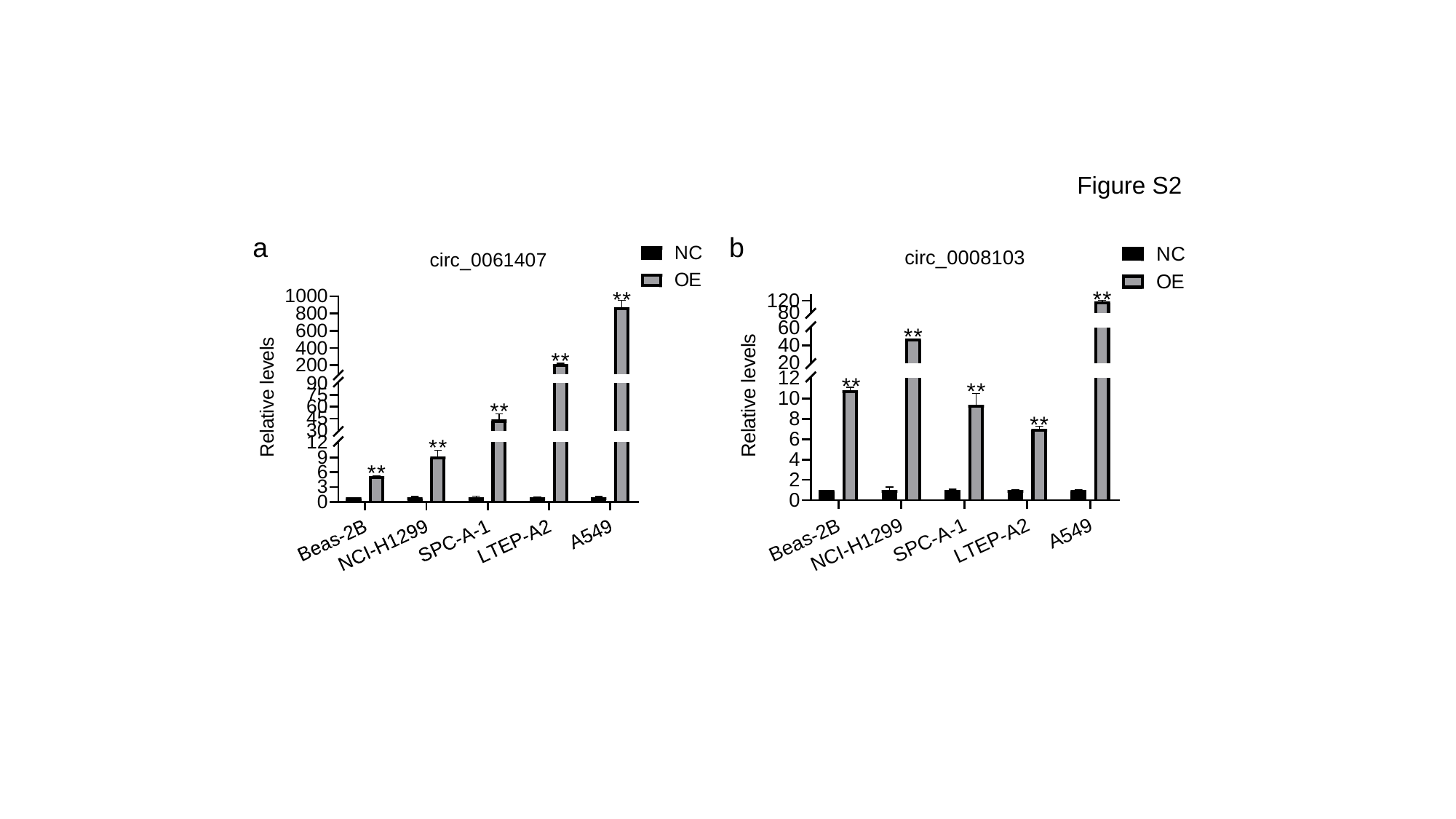

Figure S2
a
b

Supplement: Supplementary file 2 — Additional file 2: Fig. S2 The transfection efficiency of circRNA overexpression plasmids. a, b The transfection efficiency of circ_0061407 and circ_0008103 overexpression plasmids in Beas-2B and lung cancer cells. **P < 0.01. [file 12967_2024_5215_MOESM2_ESM.pptx]
