# Supplementary material for: Exosome-transported circ_0061407 and circ_0008103 play a tumour-repressive role and show diagnostic value in non-small-cell lung cancer
Source: J Transl Med. 2024 May 6;22:427. doi: 10.1186/s12967-024-05215-6 (PMC11071259; doi:10.1186/s12967-024-05215-6)
Supplement: Supplementary file 3 — Additional file 3: Fig. S3 The effect of exosome-transported circ_0061407 and circ_0008103 on recipient cells. a, e The proliferative capacity of Beas-2B cells was evaluated using the CCK-8 assay. b, f Wound healing assay was used to assess the migratory capacity of Beas-2B cells. c, g Transwell assay was performed to evaluate the migration and invasion abilities of Beas-2B cells. d, h Colony formation assay was used to detect the clone formation ability of Beas-2B cells. **P < 0.01. [file 12967_2024_5215_MOESM3_ESM.pptx]

## Slide 1
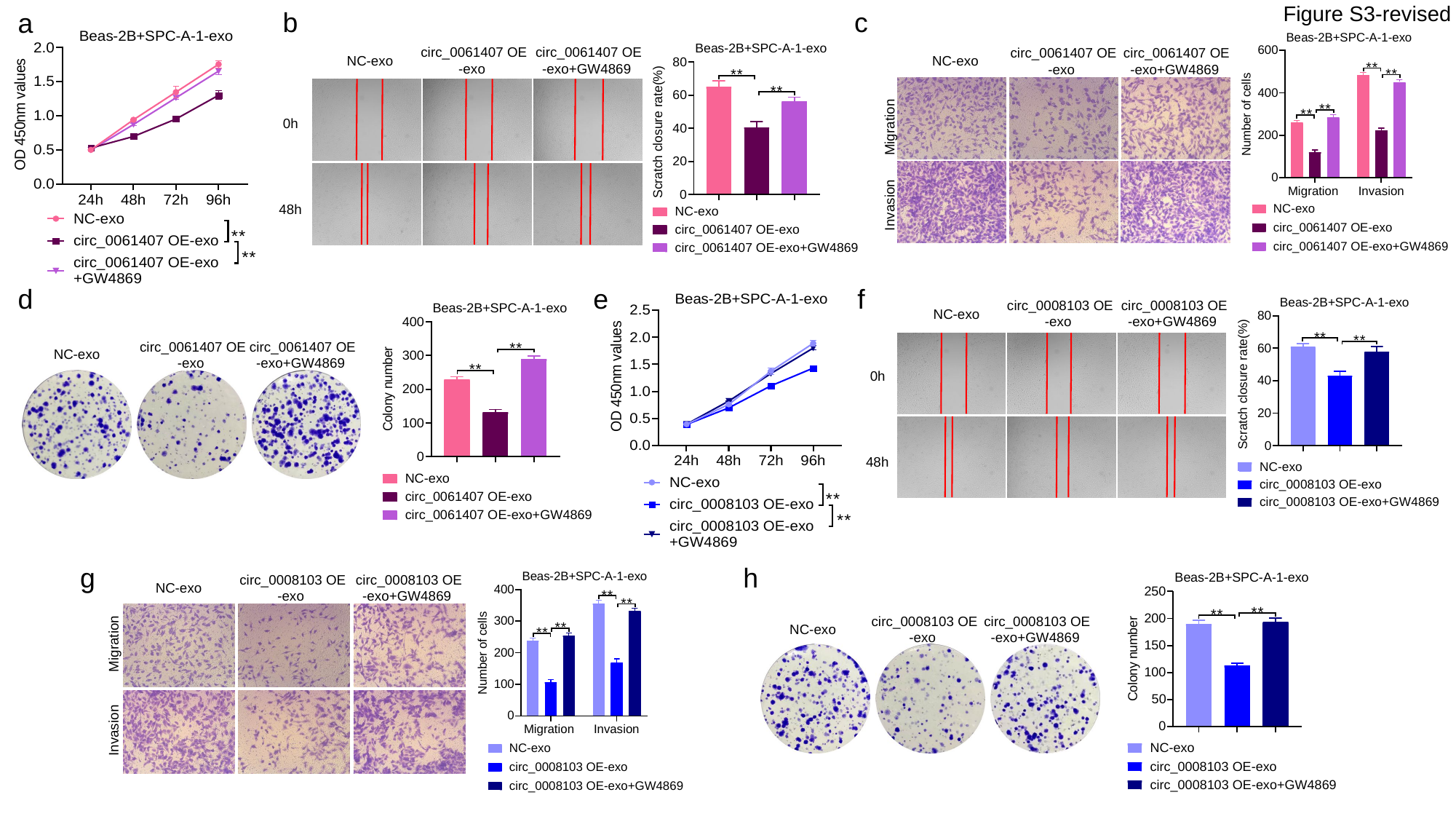

b
c
Figure S3-revised
a
 circ_0061407 OE
-exo
 circ_0061407 OE
-exo+GW4869
NC-exo
0h
48h
 circ_0061407 OE
-exo
 circ_0061407 OE
-exo+GW4869
NC-exo
Migration
Invasion
d
e
f
 circ_0008103 OE
-exo
 circ_0008103 OE
-exo+GW4869
NC-exo
0h
48h
 circ_0061407 OE
-exo
 circ_0061407 OE
-exo+GW4869
NC-exo
g
h
 circ_0008103 OE
-exo
 circ_0008103 OE
-exo+GW4869
NC-exo
Migration
Invasion
 circ_0008103 OE
-exo
 circ_0008103 OE
-exo+GW4869
NC-exo
