# Supplementary material for: Exosome-transported circ_0061407 and circ_0008103 play a tumour-repressive role and show diagnostic value in non-small-cell lung cancer
Source: J Transl Med. 2024 May 6;22:427. doi: 10.1186/s12967-024-05215-6 (PMC11071259; doi:10.1186/s12967-024-05215-6)
Supplement: Supplementary file 4 — Additional file 4: Fig. S4 MiRNAs interacted with circ_0061407 and circ_0008103. a, b Using the Pita (http://genie.weizmann.ac.il/pubs/mir07/mir07_data.html), miRanda (http://www.microrna.org/microrna/home.do), and Targetscan databases (http://www.targetscan.org/), a Venn diagram was created to show the numbers of the miRNAs that were predicted to interact with circ_0061407 and circ_0008103. c TCGA cohort (TCGA-LUAD/LUSC dataset) shows the levels of RXRB in normal (n = 108) and lung tumour (n = 1041) tissues. **P < 0.01. [file 12967_2024_5215_MOESM4_ESM.pptx]

## Slide 1
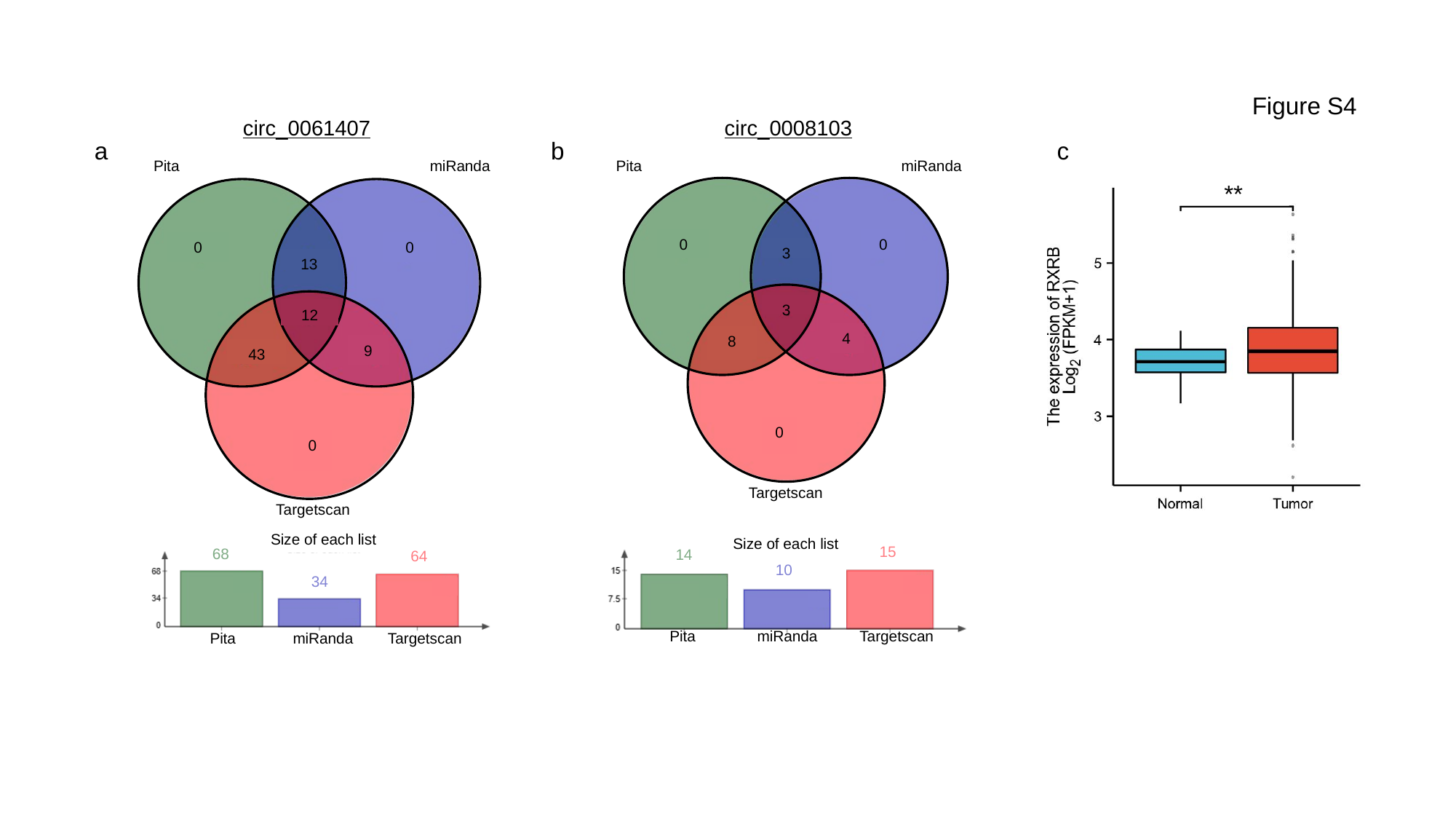

Figure S4
circ_0061407
circ_0008103
a
b
c
Pita
miRanda
Targetscan
0
0
13
12
9
43
0
Size of each list
68
64
34
Pita
miRanda
Targetscan
Pita
miRanda
Targetscan
0
0
3
3
4
8
0
Size of each list
15
14
10
Pita
miRanda
Targetscan
**
