# Supplementary material for: Exosome-transported circ_0061407 and circ_0008103 play a tumour-repressive role and show diagnostic value in non-small-cell lung cancer
Source: J Transl Med. 2024 May 6;22:427. doi: 10.1186/s12967-024-05215-6 (PMC11071259; doi:10.1186/s12967-024-05215-6)
Supplement: Supplementary file 5 — Additional file 5: Table S1. The sequences of primers used in the present study. [file 12967_2024_5215_MOESM5_ESM.docx]

Additional file 5: Table S1. The sequences of primers used in the present study

| CircRNA/GAPDH | Primer (5’ to 3’) | |
| --- | --- | --- |
| circ_0079557 | Forward | AGAGGTGGCATCTGTGAACTG |
|  | Reverse | TGATAGAGTCAGACTTCCTTGCT |
| circ_0090080 | Forward | GGTGCAATTCCTACAGACCAGA |
|  | Reverse | GCTCTCCTGCTGGAAATCCTAA |
| circ_0001177 | Forward | CTCTCTGATTGCGATGGAAGGA |
|  | Reverse | TCAAGTGTGCATCTTCTGGCT |
| circ_0091669 | Forward | AGTGCAGCCAGTTCAAAGACT |
|  | Reverse | GGACTTCTTGATTCCTCCGCT |
| circ_0061407 | Forward | CCAGCAACCCAGACACATCA |
|  | Reverse | TAGATGTCTTCCTCGGGCTGT |
| circ_0073237 | Forward | GCAACACCACCATTTTCCCT |
|  | Reverse | AATCACTCATTCGACCTGGTAA |
| circ_0007342 | Forward | GCCCACTAGGATGTAAAATAACG |
|  | Reverse | CTCCGAGGTATTTTCAGCTTGC |
| circ_0073360 | Forward | TTCTCCTACCTCAGCCTCCT |
|  | Reverse | AACATCTGCTGCAACCTGTG |
| circ_0008315 | Forward | GAGGACAGACGGCGGACAAT |
|  | Reverse | GACTCTAGGACTGCTGGTGGA |
| circ_0120779 | Forward | CAGCAGGCCAGGAAAGATTT |
|  | Reverse | GCAAGACTTTCCAACCCCTG |
| circ_0002711 | Forward | CAGAGCCCAGGTCCTGTTAA |
|  | Reverse | TCTGGCTGTGTTTCTCCCAA |
| circ_0029309 | Forward | CGGCAAGAGCCAGAAACCAG |
|  | Reverse | GGTCATAGCGCTGGCAGAAC |
| circ_0007221 | Forward | GTCTGCCTCTCCTTCCAGTT |
|  | Reverse | AACTCCAACAGCATCCAGGA |
| circ_0066443 | Forward | CGGCGTGTTGTTGTATGTCA |
|  | Reverse | TAGTGCACTCTCCCCAGTTG |
| circ_0003057 | Forward | GCAGCCCACATCAAGAAGTT |
|  | Reverse | GCCATTGCGTCCATGAAAGG |
| circ_0084648 | Forward | TCTCTTGGCAGGAATCTTCAGG |
|  | Reverse | TCTCTTGGCAGGAATCTTCAGG |
| circ_0008803 | Forward | AGGAGCTCAAGCGCATTTGTA |
|  | Reverse | TGCCCAGCAGAAGATGTGTC |
| circ_0122641 | Forward | TAGTGCCAGTGTTCAGAGGG |
|  | Reverse | ACTTGAGCCAGTACAGCCTT |
| circ_0076710 | Forward | GTTCTTCGCCATGGGATGGG |
|  | Reverse | ATGGCTCTGGCTGACTTGAC |
| circ_0008938 | Forward | GGCCAAACTCCTGGACAGTAT |
|  | Reverse | AGTGGCTGCTGCAAATTGTT |
| circ_0043110 | Forward | CAGACACAGATCCAGTTTCAGC |
|  | Reverse | TTCTCCAAGAGTCACTTCTCCT |
| circ_0045861 | Forward | TCAACCGCTACCTCCTCAAG |
|  | Reverse | ACTGGGCTGGGAAGAGTTAAG |
| circ_0008103 | Forward | GCCCAGTCAAGACACTGTTG |
|  | Reverse | AGCATTTGGCAGCTTTGCTAAT |
| GAPDH | Forward | GGGGCTCTCCAGAACATCATCC |
|  | Reverse | ACGCCTGCTTCACCACCTCTT |
