# Supplementary material for: Exosome-transported circ_0061407 and circ_0008103 play a tumour-repressive role and show diagnostic value in non-small-cell lung cancer
Source: J Transl Med. 2024 May 6;22:427. doi: 10.1186/s12967-024-05215-6 (PMC11071259; doi:10.1186/s12967-024-05215-6)
Supplement: Supplementary file 6 — Additional file 6: Table S2. Antibodies used for western blotting. [file 12967_2024_5215_MOESM6_ESM.docx]

Additional file 6: Table S2. Antibodies used for western blotting

| Antibody | Company | Cat. No. | Species | Dilution |
| --- | --- | --- | --- | --- |
| CD9 | ABclonal | A19027 | Rabbit | 1:1000 |
| CD63 | ABclonal | A19023 | Rabbit | 1:1000 |
| TSG101 | ABclonal | A2216 | Rabbit | 1:1000 |
| CD81 | ABclonal | A4863 | Rabbit | 1:1000 |
| Calnexin | ABclonal | A4846 | Rabbit | 1:1000 |
