# Supplementary material for: Exosome-transported circ_0061407 and circ_0008103 play a tumour-repressive role and show diagnostic value in non-small-cell lung cancer
Source: J Transl Med. 2024 May 6;22:427. doi: 10.1186/s12967-024-05215-6 (PMC11071259; doi:10.1186/s12967-024-05215-6)
Supplement: Supplementary file 7 — Additional file 7: Table S3. The top 46 circRNAs with the biggest log2 fold change values and the smallest P-values in circRNA microarray analysis. [file 12967_2024_5215_MOESM7_ESM.docx]

Additional file 7: Table S3. The top 46 circRNAs with the biggest log_2_ fold change values and the smallest P-values in circRNA microarray analysis

| CircRNA | P-Value | Log_2_ fold change value |
| --- | --- | --- |
| hsa_circ_0079557 | 4.31E-48 | 9.351807143 |
| hsa_circ_0090080 | 4.33E-48 | 9.351732554 |
| hsa_circ_0001177 | 1.18E-43 | 9.172853627 |
| hsa_circ_0091669 | 1.71E-43 | 9.165969888 |
| hsa_circ_0061407 | 8.17E-42 | 9.093424995 |
| hsa_circ_0073237 | 1.16E-39 | 8.996227387 |
| hsa_circ_0007342 | 1.40E-39 | 8.99247057 |
| hsa_circ_0073360 | 2.27E-37 | 8.886918876 |
| hsa_circ_0008315 | 3.61E-37 | 8.877012707 |
| hsa_circ_0120779 | 6.27E-37 | 8.865141035 |
| hsa_circ_0002711 | 4.65E-35 | 8.770004261 |
| hsa_circ_0029309 | 5.06E-35 | 8.768086964 |
| hsa_circ_0007221 | 5.80E-35 | 8.76498287 |
| hsa_circ_0066443 | 7.97E-33 | 8.649716446 |
| hsa_circ_0003057 | 7.97E-33 | 8.649716446 |
| hsa_circ_0084648 | 1.20E-32 | 8.639752548 |
| hsa_circ_0008803 | 1.33E-32 | 8.637287565 |
| hsa_circ_0122641 | 2.26E-30 | 8.508230554 |
| hsa_circ_0076710 | 2.84E-30 | 8.502274457 |
| hsa_circ_0008938 | 3.28E-30 | 8.498474176 |
| hsa_circ_0043110 | 3.72E-30 | 8.495202583 |
| hsa_circ_0045861 | 5.58E-30 | 8.484529345 |
| hsa_circ_0008103 | 5.40E-28 | 8.360075401 |
| hsa_circ_0097011 | 2.17E-62 | -8.86914662 |
| hsa_circ_0021553 | 1.13E-61 | -8.848309265 |
| hsa_circ_0079467 | 1.34E-58 | -8.756421643 |
| hsa_circ_0047744 | 2.54E-58 | -8.747878711 |
| hsa_circ_0136274 | 1.03E-54 | -8.633515835 |
| hsa_circ_0136108 | 1.15E-54 | -8.631976628 |
| hsa_circ_0070389 | 3.63E-52 | -8.548371041 |
| hsa_circ_0071616 | 4.05E-52 | -8.546715401 |
| hsa_circ_0126418 | 1.25E-49 | -8.459509098 |
| hsa_circ_0082688 | 4.01E-49 | -8.441317922 |
| hsa_circ_0085363 | 7.40E-49 | -8.431640285 |
| hsa_circ_0129039 | 4.01E-48 | -8.404733136 |
| hsa_circ_0112887 | 8.62E-48 | -8.392440263 |
| hsa_circ_0128384 | 5.97E-47 | -8.360951521 |
| hsa_circ_0003141 | 1.92E-45 | -8.30312135 |
| hsa_circ_0062983 | 3.75E-45 | -8.291732535 |
| hsa_circ_0116732 | 5.21E-45 | -8.286150366 |
| hsa_circ_0109432 | 6.11E-45 | -8.283425551 |
| hsa_circ_0125683 | 2.41E-44 | -8.259772049 |
| hsa_circ_0003321 | 9.67E-44 | -8.235571321 |
| hsa_circ_0126873 | 2.24E-43 | -8.220798034 |
| hsa_circ_0125684 | 1.32E-42 | -8.189136742 |
| hsa_circ_0008068 | 2.71E-42 | -8.17614235 |
