# Supplementary material for: Exosome-transported circ_0061407 and circ_0008103 play a tumour-repressive role and show diagnostic value in non-small-cell lung cancer
Source: J Transl Med. 2024 May 6;22:427. doi: 10.1186/s12967-024-05215-6 (PMC11071259; doi:10.1186/s12967-024-05215-6)
Supplement: Supplementary file 8 — Additional file 8: Table S4. The comparison of the relative levels of serum exosomal circ_0008103, circ_0061407, and traditional tumour markers with clinical parameters of NSCLC by Mann–Whitney U test [file 12967_2024_5215_MOESM8_ESM.docx]

Additional file 8: Table S4. The comparison of the relative levels of serum exosomal circ_0008103, circ_0061407, and traditional tumour markers with clinical parameters of NSCLC by Mann–Whitney U test

| Clinical data Factors | LUSC vs. LUAC | N0 vs. N1-3 | M0 vs. M1 | Stage I-II vs. III-IV | ≤5cm vs. >5cm |
| --- | --- | --- | --- | --- | --- |
| circ_0008103 | P=0.572 | P=0.787 | **P<0.001** | **P<0.001** | P=0.627 |
| circ_0061407 | P=0.926 | P=0.922 | **P=0.003** | P=0.073 | P=0.670 |
| CEA | P=0.243 | P=0.870 | P=0.902 | P=0.554 | P=0.527 |
| NSE | P=0.183 | P=0.104 | P=0.247 | P=0.432 | P=0.285 |
| CYFRA21-1 | P=0.483 | **P=0.027** | P=0.733 | **P=0.011** | P=0.288 |
