# Supplementary material for: Exosome-transported circ_0061407 and circ_0008103 play a tumour-repressive role and show diagnostic value in non-small-cell lung cancer
Source: J Transl Med. 2024 May 6;22:427. doi: 10.1186/s12967-024-05215-6 (PMC11071259; doi:10.1186/s12967-024-05215-6)
Supplement: Supplementary file 9 — Additional file 9: Table S5. The genes enriched in the NSCLC pathway (related to circ_0061407) and those enriched in the small cell lung cancer pathway (related to circ_0008103) [file 12967_2024_5215_MOESM9_ESM.docx]

Additional file 9: Table S5. The genes enriched in the NSCLC pathway (related to circ_0061407) and those enriched in the small cell lung cancer pathway (related to circ_0008103)

| circRNA | Target gene |
| --- | --- |
| circ_0061407 | BCL2 antagonist/killer 1 (BAK1) |
|  | EMAP like 4 (EML4) |
|  | mitogen-activated protein kinase kinase 1 (MAP2K1) |
|  | ret proto-oncogene (RET) |
|  | retinoid X receptor beta (RXRB) |
|  | kinesin family member 5A (KIF5A) |
|  | Ras association domain family member 1 (RASSF1) |
|  | serine/threonine kinase 4 (STK4) |
|  | cyclin dependent kinase 4 (CDK4) |
|  | protein kinase C beta (PRKCB) |
| circ_0008103 | cyclin dependent kinase inhibitor 2B (CDKN2B) |
|  | collagen type IV alpha 2 chain (COL4A2) |
|  | RB transcriptional corepressor 1 (RB1) |
|  | retinoid X receptor beta (RXRB) |
|  | integrin subunit alpha 6 (ITGA6) |
|  | phosphoinositide-3-kinase regulatory subunit 1 (PIK3R1) |
|  | RELA proto-oncogene (RELA) |
|  | growth arrest and DNA damage inducible gamma (GADD45G) |
|  | TNF receptor associated factor 1 (TRAF1) |
|  | integrin subunit alpha 3 (ITGA3) |
